# Non-malignant epithelial cells preferentially proliferate from nasopharyngeal carcinoma biopsy cultured under conditionally reprogrammed conditions

**DOI:** 10.1038/s41598-017-17628-z

**Published:** 2017-12-12

**Authors:** Fenggang Yu, Yanan Lu, Lin Tao, Yan-Yi Jiang, De-Chen Lin, Lingzhi Wang, Fredrik Petersson, Hironori Yoshiyama, Phillip H. Koeffler, Boon-Cher Goh, Kwok Seng Loh

**Affiliations:** 1 Department of Otolaryngology, Yong Loo Lin School of Medicine, National University of Singapore, Singapore, Singapore; 2 Cancer Science Institute of Singapore, National University of Singapore, Singapore, Singapore; 3 Department of Pathology, Yong Loo Lin School of Medicine, National University of Singapore, Singapore, Singapore; 4 Department of Microbiology, Shimane University Faculty of Medicine, 89-1 Enyacho, Izumo City, Shimane 693-8501 Japan; 5 Head & Neck Tumor group, National Cancer Institute of Singapore, Head & Neck Surgery, NUHS, Singapore, Singapore

## Abstract

Nasopharyngeal carcinoma (NPC) is an invasive cancer with particularly high incidence in Southern China and Southeast Asia. The study of NPC is greatly hampered by the lack of reliable cell lines due to the loss of EBV genome and HeLa cell contamination. Conditional reprogramming (CR) cell culture technique has been reported for rapid and efficient establishment of patient‐derived normal and tumor cell cultures. The purpose of this study was to assess this method to culture NPC patient‐derived primary tumor cells. Using CR protocol, we demonstrated that epithelial cells could be efficiently cultured from normal (70%) and cancerous nasopharyngeal (46%) biopsies. However, by comparing with original tumors in terms of mutation and methylation profiles, epithelial cells derived from cancerous biopsy represented non‐malignant cells. Further, they exhibited stem‐like characteristics based on their cell surface proteins and could differentiate into pseudostratified epithelium in an air–liquid interface culture system. We conclude that CR method is a highly selective and useful method for growing non‐malignant nasopharyngeal epithelial cells.

## Introduction

Nasopharyngeal carcinoma (NPC) is a common cancer in endemic regions such as Southern China and South East Asia^1^. NPC is very sensitive to radiotherapy at early stage, but current treatment is still associated with relapse in about 25% of patients^2^.

Undifferentiated NPC is consistently associated with Epstein-Barr virus (EBV) infection^3^. Immortalized cancer cell lines and xenografts have been used widely for the study of NPC tumor biology and testing of new therapies. However, the majority of these cell lines cannot maintain the EBV episome during continuous culture^4^. Moreover, widespread HeLa cell contamination has been documented in many NPC cell lines^5^. These two reasons make the study of tumor biology in NPC using cell lines unreliable and possibly not representative. It is therefore very necessary to develop new preclinical models for research and translation into treatment, such as primary tumor cell cultures.

Liu *et al*. reported a “conditionally reprogramed cells” method of culturing both normal and tumor epithelial cells. It uses a Rho kinase inhibitor (ROCKi) Y-27632 and irradiated mouse 3T3 fibroblast feeder cells^6^. If this method is applicable to NPC, it would represent a significant technical advancement in the establishment of primary tumor cultures from limited patient biopsy specimens. Thus the aim of this study was to assess this method of establishing primay tumor cell cultures from NPC biopsy.

## Results

### Establishment of patient-derived primary epithelial cell cultures

Ninety-two surgical biopsies were harvested and used for cell culture derivation in this study. These included 10 non-cancerous, 7 recurrent and 75 primary NPC samples. The main demographics and clinical parameters of the patients are summarized in Table 1. Of 82 primary and recurrent NPC patients, 68% were Chinese, 24% were Malay, 1% were Indian and 7% were others. Peak age of incidence was in the fifth decade (median age is 51.48 years), with the male/female ratio being 3.34:1. A total of 77% primary patients were at either stage III or IV.

**Table 1:** Patient’s characteristics and the success rate of epithelial cell culture.

Patient	Epithelial cell culture		*p-value* (univariate)
	Growth	No growth	
NPC			
Age(years)			*0.2197*
Median(range)	50(32~74)	53(38~76)	
Sex			*0.1753*
Male *(n* = *74)*	29/74 (45%)	35/74(55%)	
Female *(n* = *18)*	9/18 (50%)	9/18(50%)	
Stage			*0.1682*
I-II *(n* = *17)*	7/17(41%)	10/17(59%)	
III (n = *19*)	8/19(42%)	11/19(58%)	
IV (n = 39)	20/39(51%)	19/39(49%)	
Recurrent *(n* = *7)*	3/7(43%)	4/7(57%)	
Non-NPC			*N.A*
*(n* = *10)*	7/10(70%)	3/10(30%)	

N.A = not applicable.

**Figure 1:**
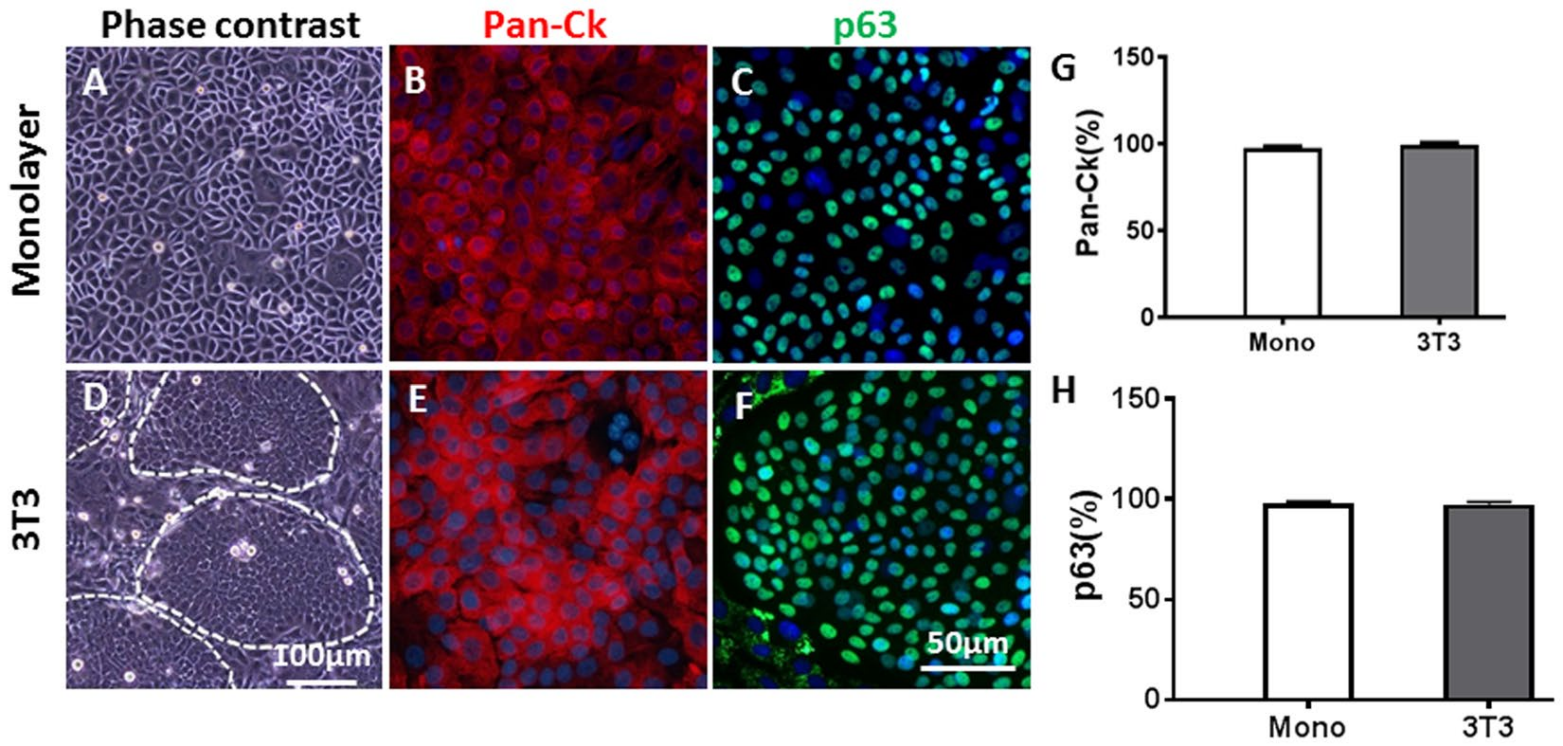
Morphology and marker expression in nasopharyngeal epithelial cell cultures. Representative microscopic images depicting cells in monolayer (**A**) and cells with 3T3 feeder layer, on which colonies formed (**D**). Expression of Pan‐CK and p63 determined by immunofluorescence (**B**,**C**,**E** and **F**).Percent positive cells plotted in bar graphs (**G** and **H**, mean ± SD, n = 3).

Cell cultures were successfully established by monolayer on plastic surfaces or by seeding on 3T3 fibroblast feeder layer. Established cultures exhibited a typical polygonal, cuboidal to slightly spindle epithelial appearance. These cells were more compact and homogeneous when cultured on a feeder layer (Fig. 1A and D). Identity of the cells was confirmed by the expression of the epithelial cell marker pan Cytokeratin (pan-CK) and basal epithelial marker p63 (Fig. 1B,C,E and F). The epithelial purity was greater than 99% (Fig. 1G and H).

The overall success rate of establishment of cell cultures including both non-cancerous and cancerous specimens was 49%, while the separate rates for non-cancerous and cancerous specimens were 70% and 46%, respectively. For establishment of cultures from cancerous specimens, no significant differences could be attributed to parameters such as age (p = 0.2197), gender (p = 0.1753) and stage of disease (p = 0.1682) (Table 1).

### Feeder cells promote growth of primary epithelial cells

**Figure 2:**
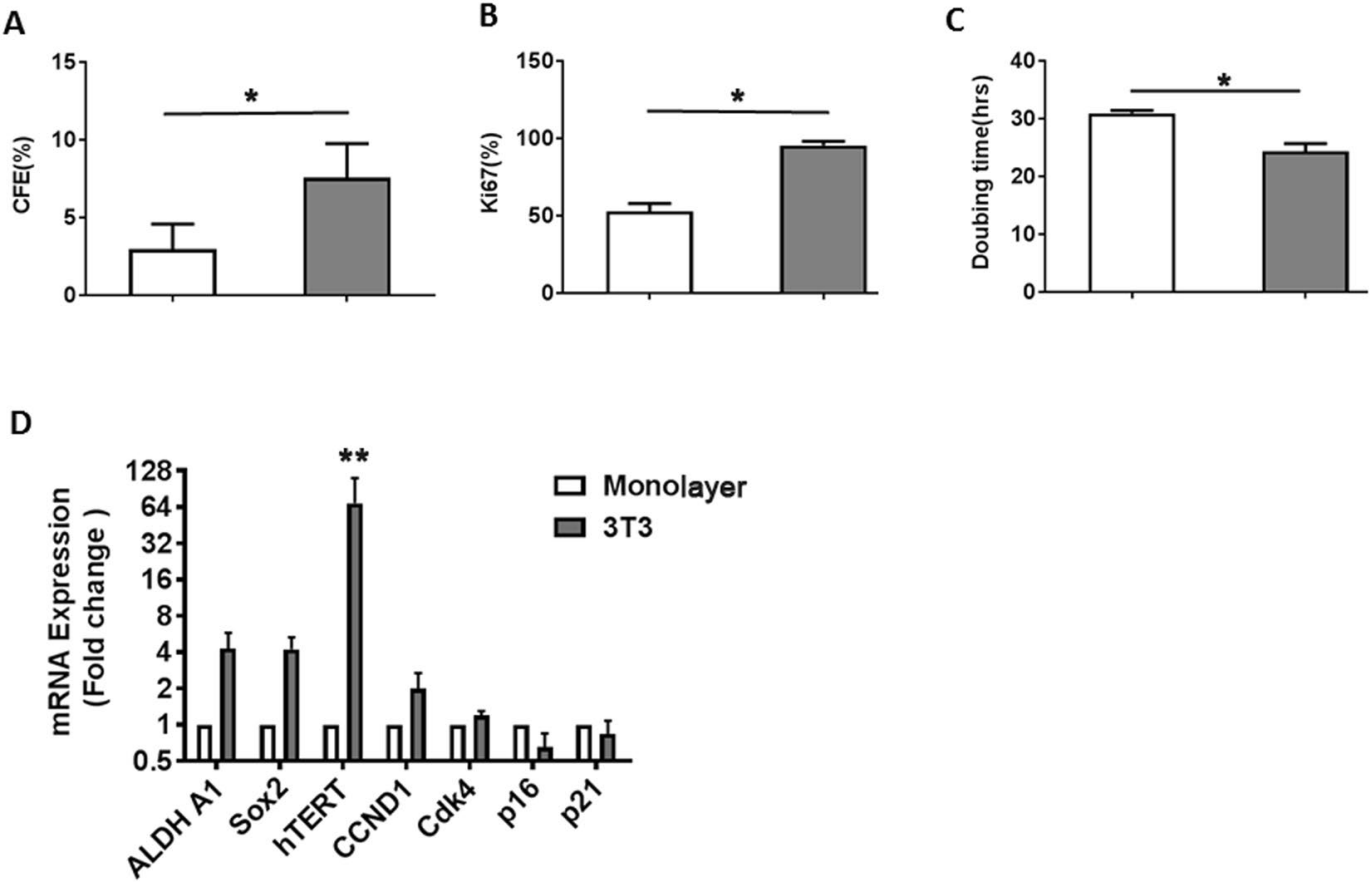
Cell growth kinetics and gene expression analysis. CFE, expression of Ki67 and doubling time were examined in monolayer cells and cells on 3T3 feeder layer (**A**–**C**). (**D**) Gene expression of cells analyzed by qPCR and the data were normalized to the housekeeping gene GAPDH. The results are presented as the fold change of cells grown on 3T3 feeder layer relative to monolayer cells (mean ± SD, n = 3). Student’s t test, *P < 0.05; **P < 0.01.

To determine the contributions of feeder cells to the expansion of primary nasopharyngeal epithelial cells, we assayed the growth kinetics of the cells under two culture conditions: 3T3 feeder cells and monolayer culture. 3T3 feeder cultures produced significantly more colonies with 7.6% of colony-formation efficiency (CFE)) than monolayer cultures (3.0% of CFE, Fig. 2A-C). Immunocytochemistry for ki67 showed 96% and 53% of total cells undergoing proliferation using 3T3 feeder layer versus monolayer conditions respectively. The doubling time of cells growing on 3T3 feeder layer versus cells on monolayer was 25 hours versus 31 hours respectively; the difference was statistically significant (p = 0.041). The monolayer cells usually became senescent within 2 to 3 passages, whereas the feeder layer could extend the cell lifespan to 10~15 passages on average.

RNA level of the cancer stem cells markers (ALDH1A1 and Sox2) and the cell cycle marker CDK1 were upregulated about 1.7~3-fold for cells on feeders (Fig. 2D), and the CDK inhibitor p16 was downregulated about 2.5-fold for the cells cultured on 3T3 feeder layer compared to monolayer cells, whereas p21 was slightly down regulated. Compared with cells grown as monolayer, hTERT activity was dramatically induced in cells growing on the feeder layer, and by passage 9, cells on the 3T3 feeders exhibited a 300-fold increase in hTERT expression as compared to cells of monolayer.

### Primary epithelial cells lack mutations found in the original tumor specimens

Although the primary nasopharyngeal cells express epithelial cell markers, we would like to know whether they are derived from malignant tissue. We previously determined the mutational landscape of 128 NPC cases using whole-exome and targeted deep sequencing^7^. Five NPC samples with the most frequent mutations were grown *in vitro* as described above, and targeted sequencing was performed on these cultured cells. No mutations were found in these cells except for two cases (FG030 and FG014). The mutant genes in these two cell cultures were 5 and 1 respectively, while the number of mutant genes in the matched NPC samples was 9 and 19 respectively (Table 2).

**Table 2:** Mutation concordance.

Cell cultures	Mutations in original biopsies	Concordance (%)
FG030	**TLR6, EVC2**, ASNS, DST, ARID2, LIMA1, PRDM2*, ROR1, CEP192	5/9
FG014	MYOT,ARHGAP26,MUC17,USP4,**ATMIN**,ZNF16 CRTC3,FCRL1,CEACAM3,PCDP1,ESRP1,SUGT1 NPRL2,ZNF777,CNN1,ATP4A,SEMA3B,PSIP1,LOXL2	1/19
FG060	MLL2,TSHZ3,MLL3, FAT1,NOTCH3	0/5
FG010	TE2	0/1
FG028	4q31 (FBXW7)	0/1

*Frameshift mutation; Matched mutations shown in block letter.

### Non-malignant epithelial cells are capable of multipotent differentiation

**Figure 3:**
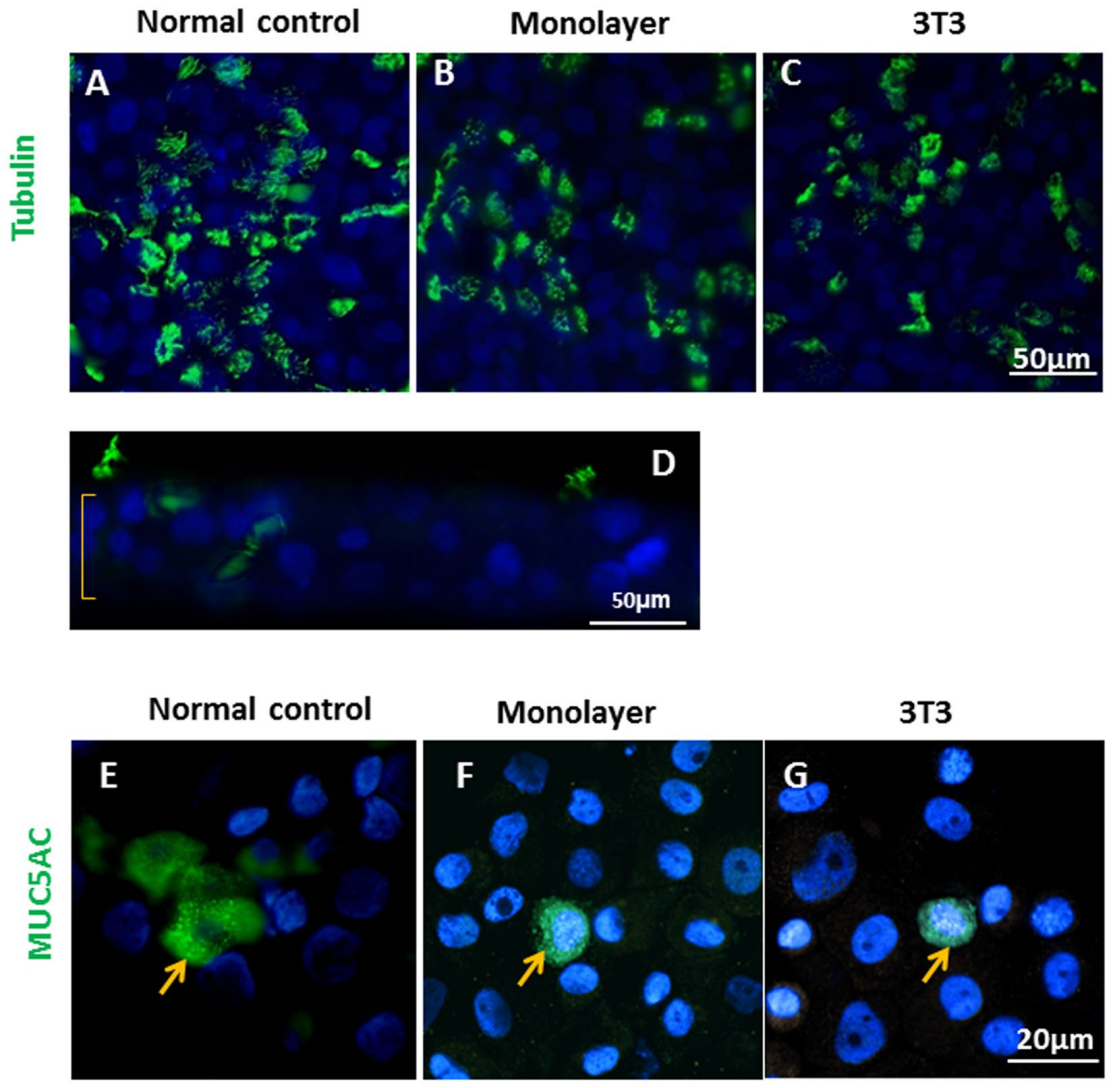
Primary cell cultures differentiated into pseudostratified epithelium at the air‐liquid interface. On‐Transwell staining (surface view) revealed clusters of cilia (β IV tubulin positive, green) on the ciliated cells differentiated from cell cultures of non-cancerous biopsy (**A**), cancerous biopsy as monolayer (**B**) and cancerous biopsy on 3T3 feeder layer(**C**). Representative image of multiple layer structure of pseudostratified epithelium differentiated from cell cultures of cancerous biopsy on 3T3 was shown in sagittal view (**D**). Differentiation into MUC5AC positive goblet cells was demonstrated as well (**E,F,G**).

Nasopharynx consists of both airway epithelium and squamous cell epithelium^8^. The non-malignant cells are believed to retain the differentiation ability, which is one of the features separating it from malignant cells^9^. To examine the differentiation ability, cells were placed in air-liquid interface (ALI) culture conditions. The transepithelial electrical resistance (TEER), an index of tight junction formation, was measured at day 21, when beating cilia usually starts to appear. The average TEER was 2000 Ω × cm^2^, suggesting the cells were capable of polarizing and forming tight junctions under ALI culture condition. Differentiation capacity was analyzed at the histological level by day 30 cross-sections of Transwell inserts. Pseudostratified ciliated epithelium had developed (Fig. 3). Also formation of beating cilia occurred with the beat frequency ranging from 7 to 9 khz, which is comparable to that of normal controls. Immunofluorescent staining demonstrated that, on average, 9.3% of β IV tubulin positive ciliated cells and 3.1% of mucin secreting cells were present in the day 30 ALI epithelium. About 30% of the total cells remained positive for the basal cell marker p63, indicating a proportion of the epithelial cells did not differentiate (Table 3). Functionally, these cells maintained pluripotent properties allowing them fully differentiate into pseudostratified ciliated epithelium.

**Table 3:** Nasopharyngeal epithelial cells differentiate into pseudostratified epithelium.

Condition	Percentage of cells (%)		
Marker	P63	β IV Tubulin	MUC5AC
Monolayer	29.6 ± 13.2	7.9 ± 5.7	4.3 ± 1.2
3T3	33.8 ± 20.6 2.	7 ± 0.7	4.8 ± 1.8

## Discussion

Liu *et al*. first reported the conditional reprogramming culture methods in 2012, which was subsequently summarized in 2017^6,10^. They reported that patient-derived cell cultures can be rapidly and efficiently established from both normal and tumor biopsies. Primary cultures of NPC are known to be notoriously difficult to establish, hence it was worthwhile trying to use CR method. Although epithelial cell proliferation was robust, the cultured cells were mostly non-malignant cells based on careful molecular and functional characterization. It contradicts Liu’s report but is similar to a study by Gao *et al*., who found CR cultures of lung cancer samples were non-malignant^11^.

**Figure 4:**
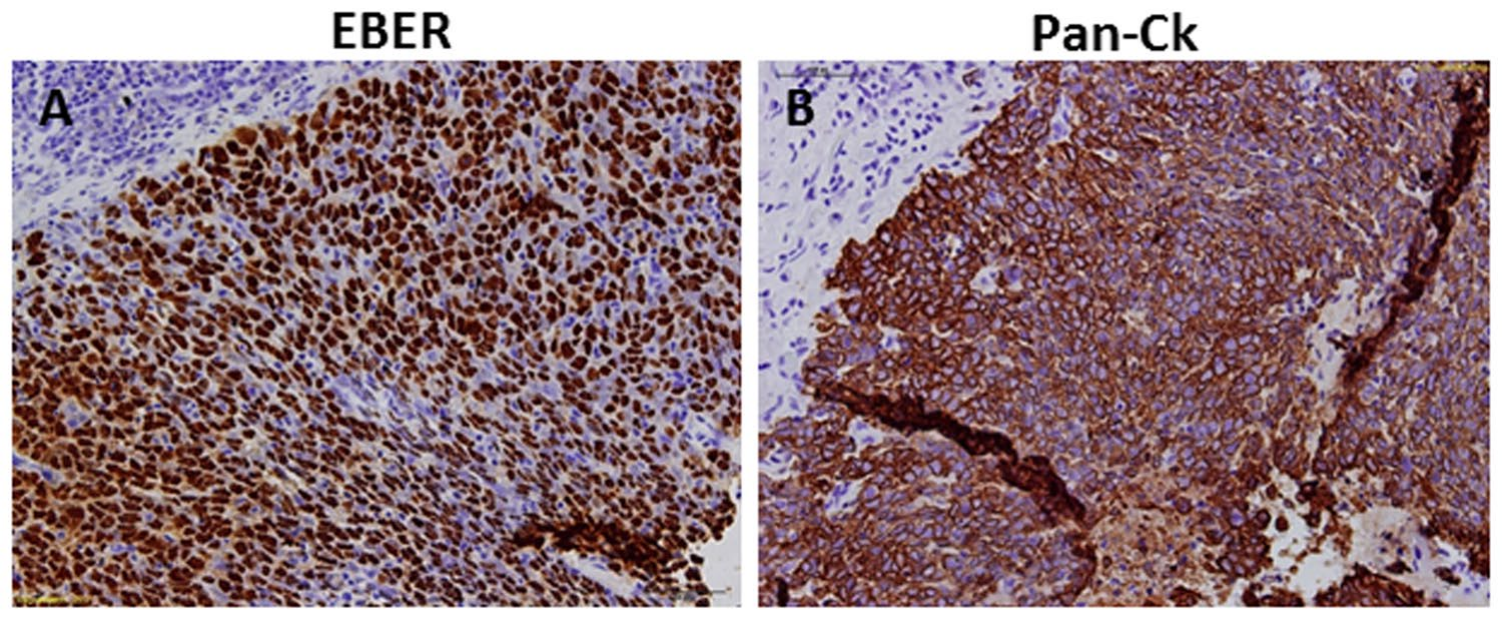
Histology and marker expression of NPC tissue FG014 (200×). Consecutive sections at 4 μm thickness were stained for expression of EBER and pan‐CK. EBER‐ISH showed an intense nuclear labeling exclusively in the tumor cells (**A**), and no staining was observed in surrounding or infiltrating lymphocytes (recognized by dense staining of hematoxylin in the small and round cell nuclear). The same group of EBER positive cells was also stained positive for pan‐CK (**B**).

The patient tissues used in this study were endoscopically biopsied from the core of tumor. The existence of tumor cells was pathologically confirmed. For example, the FG014 biopsy had more than 90% of EBER-ISH cells among pan-CK positive cells, consistent with the majority of epithelial cells of this sample were tumor cells (Fig. 4A and B). Nevertheless, the non-malignant cells grew robustly, suggesting this culture condition favours normal cell growth. Indeed, when real time PCR of EBNA1 was used to track tumor cells, EBNA1 Initially was detected in cell culture, but it diminished very quickly. NPC cells are known to lose EBV during *in vitro* culture^12^. Even if the culturing of NPC tumor cells accelerates the loss of EBV, we should still be able to detect their nucleotide mutations. However we failed to do so. The lack of mutations in cell cultures suggested that the cells growing under CR conditions were predominantly non-malignant. NPC tumors are known to have wide spread CpG genomic methylations associated with EBV infection^13,14^. Therefore we applied Illumina Infinium HumanMethylation450K array to measure genome-wide methylation changes. The cell cultures showed little methylation, further supporting the non-malignant nature of the cultured cells (data not shown).

In a previous study, the establishment of NPC cultures from C17 sample were shown facilitated by CR method, which is an EBV-positive xenograft propagated by subcutaneous passages into nude mice^15^. What makes it different from current study is that C17 is a well-established tumor xenograft assumingly consisting of pure tumor cells and no non-malignant cells to compete. In order to use this CR method, tumor tissues have to be dissociated into single cells, which may disrupt the tumor niche. Successful NPC tumor cell cultures may need retention of cell-cell contact as reported for cells from colorectal and retinoblastoma^16,17^. Our study clearly showed that CR method is not suitable for NPC culture. Derivation of primary tumor cell cultures is important for testing personalized therapies. Successful and reproducible growth of NPC tumor specimens will require modification of the current protocol or the development of new methodology.

Another limitation of this method is the use of murine 3T3 cells as feeder layer. It introduces xeno-components and confounds the interpretation of results. Viable residual 3T3 feeder cells can form carcinoma-like xenograft tumour^18,19^.

The advantage of this method is the rapid generation of  non-malignant epithelial cells without genetic manipulation, and the cells retain stem‐like properties. Indeed, these non‐malignant cells can differentiate into pseudostratified epithelium as shown here. The  non-malignant  nasopharyngeal epithelial cells could be utilized as controls in  NPC studies due to the scarcity of normal naspharyngeal tissues.

## Materials and Methods

### Biopsy collection

The present study was approved by National Healthcare Group Domain‐Specific Review Board of Singapore (Reference No.: DSRB‐B/10/337). Informed and written consent was obtained from all patients. All procedures were carried out in accordance with the approved guidelines.

One portion of nasopharyngeal biopsy specimen was sent to pathology laboratory for diagnosis. Another portion was used for nucleotide sequencing. A third portion was immediately transported to the laboratory. The specimen was washed extensively in Hank’s balanced salt solution containing penicillin, streptomycin, and amphotericin B. The specimen was digested with 10 mg/ml of Dispase II (Sigma, St. Louis, MO) at 4 °C overnight, followed by dissociation by repetitive pipetting. The digestion was stopped by adding media containing serum. The dissociated cells were washed twice and cultured either as a monolayer or co‐cultured on top of feeder cells.

### Feeder layers preparation

Mouse NIH 3T3 cells were obtained from American Type Culture Collection (ATCC, Manassas, VA) and cultured in DMEM supplemented with 10% FBS. In order to induce growth‐arrest, NIH 3T3 cells were irradiated at 30 Gy in 3 min as previously described^18^.

### NPC primary cell culture

For monolayer culture, cells were seeded in basal medium (CELLnTEC, Bern, Switzerland) in six‐well plates For co‐culture, irradiated feeder cells were pre‐plated at a cell density of 2 × 10^4^cells/cm^2^ in six‐well plates and allowed to attach for at least 2 hr. NPC cells were seeded at a density of 4 × 10^3^/cm^2^ in FG medium (Basal medium/Dulbecco’s Modified Eagle Medium(3:1(v/v)),0.02% fetal bovine serum, 10 μM of Y27632).

### Immunocytochemistry and EBER *in situ* hybridization

Immunocytochemistry was conducted as per standard protocols as previously described^18,20^. Epstein–Barr virus encoded early RNA *in situ* hybridization (EBER‐ISH) was performed according to the manufacturer’s instructions (Dako, Glostrup, Denmark).

### Doubling time and CFE

Assays of doubling time and colony forming efficiency (CFE) were conducted as previously described^21^.

### Polymerase chain reaction (PCR)

DNA and RNA were extracted from cell cultures using Qiagen AllPrep DNA/RNA Mini Kit according to manufacturer’s protocol. For cDNA synthesis, 1µg of total RNA was reversely transcribed by using a Maxima Reverse Transcriptase Kit (Thermo Scientific, Rockford, IL) based on the manufacture’s protocol. TaqMan assays (Life Technologies) were used to evaluate the target gene expression. Relative gene expression was analyzed using the 2^−ΔΔCt^ method with GAPDH as an internal reference.

### Mutation validation

Genomic DNA was isolated from tumor tissues using the Qiagen AllPrep DNA/RNA Mini Kit as described by the manufacturer. PCR was performed on the genomic DNA samples using a 50 μl of final volume containing 40~100 ng of template DNA, 2.0 μl of 10xPCR buffer, 1.5 mM MgCl_2,_ 0.5 μM of each primer, 2 units of Taq DNA polymerase (10 U/μl; Takara) with 0.2 mM dNTP. Amplifications were performed using Veriti Thermal Cycler (Thermo Fisher Scientific). In each PCR, the reaction mixtures were denatured at 95 °C for 5 min, and then subjected to 32 cycles. Each cycle consisted of 95 °C for 15 s, 55‐62 °C for 30 s, and 72 °C for 40 s to 1 min. Final extension at 72 °C for 5 min. Subsequently, amplified fragments were sequenced. Sequences were analyzed using Sequencher software (Gene Codes Corp, Ann Arbor, MI).

### Transwell differentiation

About 1 × 10^5^ NPC tumor cells were resuspended in 100 μl of B‐ALI^TM^ growth medium (Lonza, Walkersville, MD) and pipetted onto 24‐well Transwell inserts (0.4 μm pore size, Corning, NY) with 400 μl of B‐ALI^TM^ growth medium which was added to the basal chamber. On day 3 after seeding, B‐ ALI^TM^ growth medium from the apical and basal chambers was removed, and 400 μl of B‐ALI^TM^differentiation medium was added to the basal chamber only. The differentiation medium of the basal chamber was changed every other day during the 4‐week culture period. Transepithelial electrical resistance (TEER) and Ciliary beat frequency (CBF) were measured as previously described^22^. Cell types were determined by staining of cytospin preparations with indicated lineage markers: β IV tubulin for ciliated cells, MUC5AC for goblet cells and p63 for basal cells. Quantification was given in Table 3 (mean ± SD, n = 3).

### Statistics

All data were presented as a mean ± SD from a minimum of three independent experiments. Data were analyzed and plotted in GraphPad Prism.
